# New material and revision of *Melanorosaurus thabanensis*, a basal sauropodomorph from the Upper Triassic of Lesotho

**DOI:** 10.7717/peerj.1639

**Published:** 2016-02-01

**Authors:** Claire Peyre de Fabrègues, Ronan Allain

**Affiliations:** Muséum National d’Histoire Naturelle, Centre de Recherche sur la Paléobiodiversité et les Paléoenvironnements (CR2P, UMR 7207), Sorbonne Universités-MNHN, CNRS, UPMC, Paris, France

**Keywords:** Dinosauria, Sauropodomorpha, *Melanorosaurus*, *Meroktenos*, Southern Africa, Lesotho, Upper Triassic, Lower Jurassic

## Abstract

*Melanorosaurus* is a genus of basal sauropodomorph that currently includes two species from Southern Africa. In this paper, we redescribe the holotype femur of *Melanorosaurus thabanensis* from the Elliot Formation of Lesotho, as well as associated remains. The stratigraphic position of this taxon is reviewed, and it is clear that it comes from the Lower Elliot Formation being, therefore, Late Triassic in age, and not Early Jurassic as originally described. The knowledge of the anatomy of the basal sauropodomorph of Thabana Morena is enhanced by the description of six new skeletal elements from the type locality. The femur and the ilium from Thabana Morena are diagnostic and characterized by unusual proportions. The first phylogenetic analysis including both this specimen and *Melanorosaurus* is conducted. This analysis leads to the conclusion that the femur described in the original publication of *Melanorosaurus thabanensis* can no longer be referred to *Melanorosaurus*. For these reasons, we hereby create *Meroktenos* gen. nov. to encompass *Meroktenos thabanensis* comb. nov.

## Introduction

Since the description of *Thecodontosaurus* (Riley & Stutchbury, 1836) and *Plateosaurus* (Meyer, 1837), approximately 40 genera of basal sauropodomorphs (i.e., non-Sauropoda Sauropodomorpha) have been discovered worldwide. Most of them are Gondwanan forms, coming from South America (twelve genera) and Southern Africa (ten genera; see Table 1 for an exhaustive list with associated publications). The first basal sauropodomorph genus from the upper Elliot Formation of South Africa was described in 1854 by Owen and named *Massospondylus* (Owen, 1854). Since then, nine other genera based on more or less complete material have been described (some being currently regarded by some authors as nomina dubia): *Euskelosaurus* (Huxley, 1866), *Eucnemesaurus* (Van Hoepen, 1920), *Melanorosaurus* (Haughton, 1924), *Plateosauravus* (Huene, 1932) and *Blikanasaurus* (Charig, Attridge & Crompton, 1965) from the lower Elliot Formation (Table 1). *Antetonitrus* (Yates & Kitching, 2003; first assigned to the lower Elliot, it is now considered as upper Elliot, J Choiniere & B McPhee, pers. comm., 2015), *Aardonyx* (Yates et al., 2010) and *Arcusaurus* (Yates, Bonnan & Neveling, 2011) from the upper Elliot Formation, and, lately, *Sefapanosaurus* (Otero et al., 2015), for which the stratigraphic position is undetermined (Table 1). Thus far, *Massospondylus* and *Melanorosaurus* are the only genera known from both South Africa and Lesotho (Galton & Upchurch, 2004). Another basal sauropodomorph (i.e., ‘the Maphutseng dinosaur’), discovered by a team led by Paul and François Ellenberger in the 1950s, is known from Lesotho (Ellenberger & Ellenberger, 1956; Ellenberger & Ginsburg, 1966). The material, under review, was preliminarily published in 1993 (Gauffre, 1993b) and fully described in a PhD thesis (Gauffre, 1996), but has unfortunately never been published further.

**Table 1:** Elliot Formation basal sauropodomorphs. List of the 11 genera of basal sauropodomorphs from Southern Africa (10 published and ‘The Maphutseng dinosaur’) found in the lower Elliot Formation (Late Triassic) or in the upper Elliot Formation (Early Jurassic), with associated publications and sorted by date of first publication.

	Taxa	Publications
Lower Elliot Formation	*Euskelosaurus* (Huxley, 1866)	Huxley, 1866; Huxley, 1867; Seeley, 1894; Huene, 1906; Broom, 1911; Haughton, 1924; Van Heerden, 1979; Cooper, 1980
	*Eucnemesaurus* (Van Hoepen, 1920)	Van Hoepen, 1920; Van Heerden, 1979; Galton, 1985; Yates, 2007a; McPhee et al., 2015
	*Melanorosaurus* (Haughton, 1924)	Haughton, 1924; Van Heerden, 1979; Gauffre, 1993a; Van Heerden & Galton, 1997; Welman, 1999; Galton, Van Heerden & Yates, 2005; Bonnan & Yates, 2007; Yates, 2007b
	*Plateosauravus* (Huene, 1932)	Huene, 1932; Van Heerden, 1979; Yates, 2003
	*Blikanasaurus* (Charig, Attridge & Crompton, 1965)	Charig, Attridge & Crompton, 1965; Galton & Van Heerden, 1985; Galton & Van Heerden, 1998; Yates, 2008
	‘The Maphutseng dinosaur’	Ellenberger & Ellenberger, 1956; Ellenberger & Ginsburg, 1966; Gauffre, 1993b; Gauffre, 1996
Upper Elliot Formation	*Massospondylus* (Owen, 1854)	Owen, 1854; Cooper, 1981; Gow, 1990; Gow, Kitching & Raath, 1990; Sues et al., 2004; Barrett & Yates, 2005; Reisz et al., 2005; Barrett, 2009; Reisz et al., 2010; Yates & Barrett, 2010; Butler et al., 2013
	*Antetonitrus* (Yates & Kitching, 2003)	Yates & Kitching, 2003; McPhee et al., 2014
	*Aardonyx* (Yates et al., 2010)	Yates et al., 2010
	*Arcusaurus* (Yates, Bonnan & Neveling, 2011)	Yates, Bonnan & Neveling, 2011
?	*Sefapanosaurus* (Otero et al., 2015)	Otero et al., 2015

*Melanorosaurus* is considered by some authors as the only basal sauropodomorph genus found both in Triassic and Jurassic deposits from Southern Africa (Gauffre, 1993a; Galton & Upchurch, 2004). *Melanorosaurus readi* (Haughton, 1924) is known from Late Triassic-aged (Norian) deposits in the Eastern Cape and Free State Provinces, South Africa (Galton, Van Heerden & Yates, 2005). *Melanorosaurus thabanensis* was described based on an isolated right femur, as “the only Early Jurassic Melanorosauridae” (Gauffre, 1993a:653). Recently, it has transpired that six other bones ‘associated’ with the femur, and assigned the same specimen number, were discovered in the collections of the Muséum National d’Histoire Naturelle (MNHN), Paris. This material was originally collected in 1959 by a team led by François Ellenberger in the area of Thabana Morena, Lesotho (Costedoat, 1962; Ellenberger et al., 1964; Ellenberger, 1970). The purpose of the present paper is, firstly, to present a more exhaustive description of the femur already figured by Gauffre (1993a) and to describe for the first time the other elements. It is, then, to show that the assignment of this specimen to *Melanorosaurus* is no longer justified and that, after a comparison with other sauropodomorph taxa known from South Africa and Lesotho, the creation of a new generic combination is necessary.

## Material & Methods

### Geological overview and the stratigraphic origin of *Melanorosaurus thabanensis*

**Figure 1:**
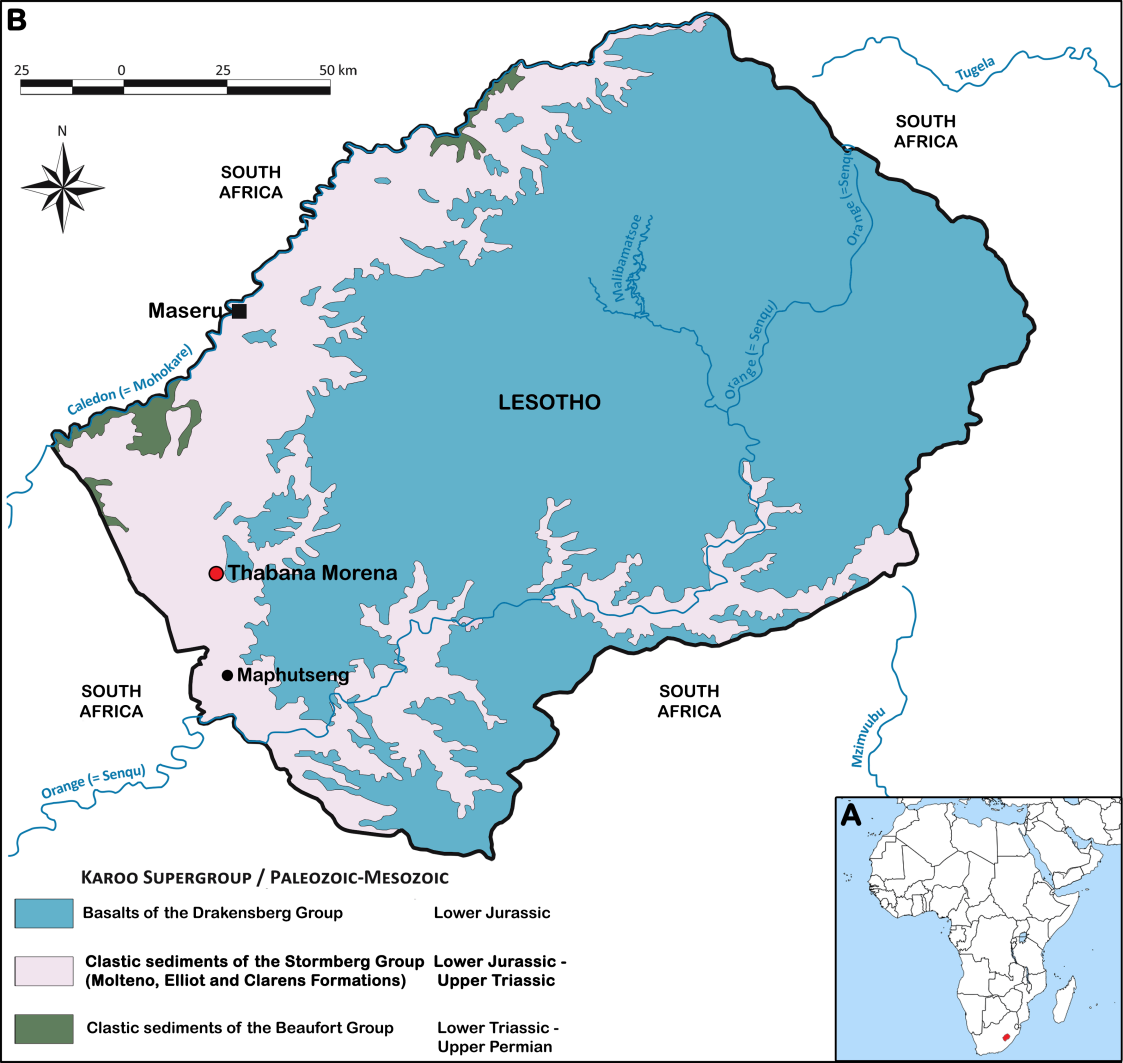
Karoo Supergroup in Lesotho (modified from Schlüter, 2008). (A) Geographical location of Lesotho. (B) Geological map of Lesotho showing the location of Thabana Morena village. The exact geographical location of the site that yielded *M. thabanensis* is unknown.

The Kingdom of Lesotho is located in the Karoo basin: it is thus exclusively made up of rocks from the Karoo Supergroup. Volcanic rocks dominate, by covering all the central and eastern part of the country, whereas sedimentary rocks crop out over the western areas of Lesotho (Schlüter, 2008) (Fig. 1).

The lower parts of the Karoo Supergroup (i.e., rocks of the Dwyka and Ecca Groups) are not exposed in Lesotho. The lowest stratigraphic layers exposed (Upper Permian) are part of the Permo-Triassic Beaufort Group. The uppermost ones are represented by the Jurassic Drakensberg Group (Schlüter, 2008). The Stormberg Group, ranging from Late Triassic (Carnian) to Early Jurassic (Pliensbachian) in age, mostly occurs on the western side of the country and in the Senqu valley (Bordy, Hancox & Rubidge, 2004a; Schlüter, 2008) (Fig. 1). The Stormberg group is subdivided into three formations, originally created by Dunn (1878), and later termed Molteno, Elliot and Clarens by the South African Commitee for Stratigraphy (1980) (Kitching & Raath, 1984).

The Elliot and Clarens Formations (historically called ‘Red Beds’ and ‘Cave Sandstone,’ respectively) are the ones where basal sauropodomorphs remains are found (Haughton, 1924; Ellenberger et al., 1964; Ellenberger, 1970; Kitching & Raath, 1984). The Elliot Formation is subdivided into lower and upper members (Bordy, Hancox & Rubidge, 2004b). The age of the boundary between these two units is not properly established (Knoll, 2005). However, the lower Elliot Formation is dated to the Late Triassic while the upper Elliot Formation is usually considered Early Jurassic in age (Olsen & Galton, 1984; Smith & Kitching, 1997; Bordy, Hancox & Rubidge, 2004a).

Thabana Morena is a hill and plateau formed by Drakensberg basalts, underlain by Stormberg sediments. Both lower and upper Elliot fossil-bearing rocks crop out in the Thabana Morena area (B Battail, pers. comm., 2014). In the original publication, Gauffre (1993a) affirmed that the material is from the upper Elliot Formation. Three years later, he argued that the material is not from the Early Jurassic but from Late Triassic (Gauffre, 1996:47), basing this on the Master thesis of Costedoat (1962). Indeed, the type material of *Melanorosaurus thabanensis* was first figured in this unpublished work, with the mention that it was found during a 1959 field trip carried out by F Ellenberger, with the help of J Fabre and L Ginsburg (Costedoat, 1962). It is also stated that the remains have been collected approximately “at the center of the Lower Red Beds, well below the *Tritylodon* Acme Zone” (Costedoat, 1962:43). This zone is at the base of the upper Elliot Formation (Ellenberger, 1970; Smith & Kitching, 1997), and the Lower Red Beds are now considered to be the equivalent of the lower Elliot Formation (Smith & Kitching, 1997; Bordy, Hancox & Rubidge, 2004a). Thus, we concur with Costedoat (1962) and Gauffre (1996) that the remains of *M. thabanensis* are of Late Triassic age and not of Early Jurassic age, in contrast to published works (e.g., Gauffre, 1993a; Galton & Upchurch, 2004; Galton, Van Heerden & Yates, 2005).

### The taxonomic status of *Melanorosaurus*

In 1924, the taxon *Melanorosaurus* was erected with, as type species, *M. readi* (Haughton, 1924). Haughton wrote “The bones consist of a tibia, a fibula, part of the pelvis, some vertebrae and metatarsals, together with a femur lying partly embedded in the overlying sandstone and the proximal half of a humerus found weathered down the slope. They are in the collection of the South African Museum (Cat. Nos. 3449, 3450).” (Haughton, 1924:429). This material, which is now numbered SAM-PK-3449 and SAM-PK-3450, represents the syntype series of *M. readi*, to the exclusion of the femur found in the higher stratigraphic layer (Haughton, 1924:433). A referred specimen (SAM-PK-3532) was also cited. Van Heerden (1979) studied the type material and assigned most of the elements except one sacral, possibly the tibia and the weathered femur, to *Euskelosaurus*. In 1997, Van Heerden & Galton (1997) referred a new specimen: NM QR1551 to *M. readi,* on the basis of similarities between the femur of this specimen and the one belonging to the syntype series. Later, Galton, Van Heerden & Yates (2005:5) stated that “many additional bones, mostly of *Plateosauravus*, were catalogued with SAM 3449 and SAM 3450 since 1924…” In the same paper, a new specimen (NM QR3314) was referred to *M. readi*. Finally, *Melanorosaurus readi* was regarded as a “Sauropodomorpha incertae sedis pending further analysis of the holotype and of all the referred specimens” (Galton, Van Heerden & Yates, 2005:32). In 2007, Yates also wrote that “[…] a lot of extraneous material has been accessioned under both of these numbers” (Yates, 2007b:11), and, according to him, the specimen NM QR1551 can be referred to *M. readi* based on the sacrum, which displays two autapomorphies showing its affiliation with *M. readi*. The specimen NM QR3314 was referred to NM QR1551 based on these two autapomorphies, and thus indirectly referred to *M. readi* (Yates, 2007b). A revised diagnosis was proposed (Yates, 2007b), based on NM QR1551 and 3314, but not on the type specimens. This raises serious doubts about the taxonomic status of *Melanorosaurus*. Recently, it was made clear that the type of *M. readi* has not been properly considered for the last 90 years, and that the modern understanding of this taxon is not based on the syntype, but on the referred specimens (Nair & Yates, 2014). In this context, the core group of bones initially described by Haughton (1924) should be reexamined in order to identify diagnostic features, and the elements supporting these features should be treated as the lectotype of *M. readi*. This question will be treated in a forthcoming paper (J Nair, pers. comm., 2014), and is outside of the scope of that study. Whether NM QR1551 and NM QR3314 are really *Melanorosaurus readi* or another taxon is something which still needs to be demonstrated after a “thorough description and evaluation of the composition of the syntype series” (Yates, 2007b:11; McPhee et al., 2015). In the rest of this paper, and awaiting a clarification regarding the status of *Melanorosaurus*, we will carry out comparisons with *M. readi* based on its syntype material (Haughton, 1924) on the one hand, and with NM QR1551 and NM QR3314 on the other hand. The syntype of *M. readi*, housed in Cape Town, as well as the specimens NM QR1551 and NM QR3314, stored in the National Museum of Bloemfontein, were examined first-hand by the senior author of this paper.

## Systematic Palaeontology

**Table:** 

Dinosauria Owen, 1842
Saurischia Seeley, 1887
Sauropodomorpha Huene, 1932
Sauropodiformes Sereno, 2005
*Meroktenos* gen. nov.

**Zoobank** urn:lsid:zoobank.org:act:D2F95159-3806-4EF8-98DF-F027E5810C1D:

Figs. 2–7.

**Type species** *Melanorosaurus thabanensis* (Gauffre, 1993a).

**Diagnosis** Same as the type and only known species.

**Etymology** From the ancient Greek μηρóς (*mēros*): femur, and $\mathrm{\kappa }\mathrm{\tau }\tilde {\mathrm{\eta }}\mathrm{\nu }$ oς (*ktênos*): animal, beast because the species was first described based only on its femur.

*Meroktenos thabanensis* (Gauffre, 1993a) comb. nov.

**Nomenclatural acts** The electronic version of this article in Portable Document Format (PDF) will represent a published work according to the International Commission on Zoological Nomenclature (ICZN), and hence the new names contained in the electronic version are effectively published under that Code from the electronic edition alone. This published work and the nomenclatural acts it contains have been registered in ZooBank, the online registration system for the ICZN. The ZooBank LSIDs (Life Science Identifiers) can be resolved and the associated information viewed through any standard web browser by appending the LSID to the prefix http://zoobank.org/. The LSID for this publication is: [urn:lsid:zoobank.org:pub:6BD17539-F024-432A-9232-B220BBBE0EDF]. The online version of this work is archived and available from the following digital repositories: PeerJ, PubMed Central and CLOCKSS.

**Table:** 

1964 “large prosauropod bones”; Ellenberger et al., 1964: 326.
1970 unnamed melanorosaurid prosauropod “Mélanorosauridé”; Ellenberger, 1970: 346.
1993 *Melanorosaurus thabanensis* Gauffre
1997 *Melanorosaurus thabanensis*; Van Heerden & Galton, 1997: 39, 40, 48, 50, Figs. 5C, I.
2004 *Melanorosaurus thabanensis*; Galton & Upchurch, 2004: 235, 251, 255.
2005 *Melanorosaurus thabanensis*; Galton, Van Heerden & Yates, 2005: 23, Figs. 1.13C, I.
2007 *Melanorosaurus thabanensis*; Yates, 2007b: 12.
2009 *Melanorosaurus thabanensis*; Barrett, 2009: 1032.
2010 *Melanorosaurus thabanensis*; Knoll, 2010: 1.

**Holotype.** MNHN.F.LES16. Material figured in the original publication (Gauffre, 1993a): right femur (MNHN.F.LES16c; Fig. 6; Tables 2 and 3) as well as associated material found in the collections: incomplete right ilium with a dorsal? neural arch ablated to the acetabulum (MNHN.F.LES16a; Fig. 4; Table 4); left pubis (MNHN.F.LES16b; Fig. 5); right metatarsal II (MNHN.F.LES16d; Fig. 7).

**Type locality.** Thabana Morena area, Mafeteng district, Lesotho. The exact geographical location of the site was not specified and, although Gauffre (1993a:648) mentions a location “4–5 km south of Thabana Morena village,” remains unknown.

**Type horizon.** Lower Elliot Formation, Upper Triassic.

**Figure 2:**
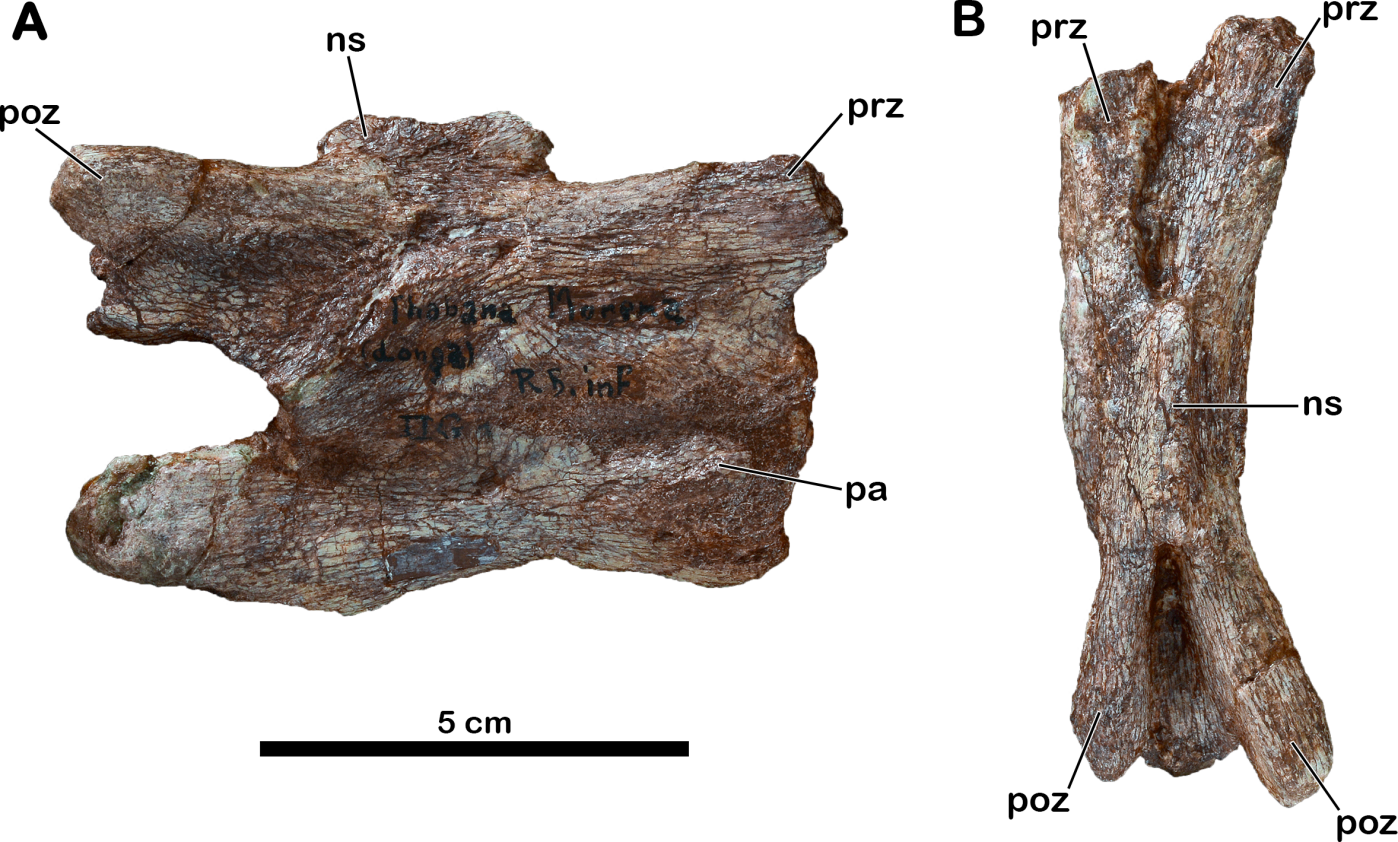
Anterior cervical vertebra of *Meroktenos*, MNHN.F.LES351a. (A) Right lateral and (B) dorsal views. ns, neural spine; pa, parapophysis; poz, postzygapophyses; prz, prezygapophyses. (Photo credit: L Cazes.)

**Figure 3:**
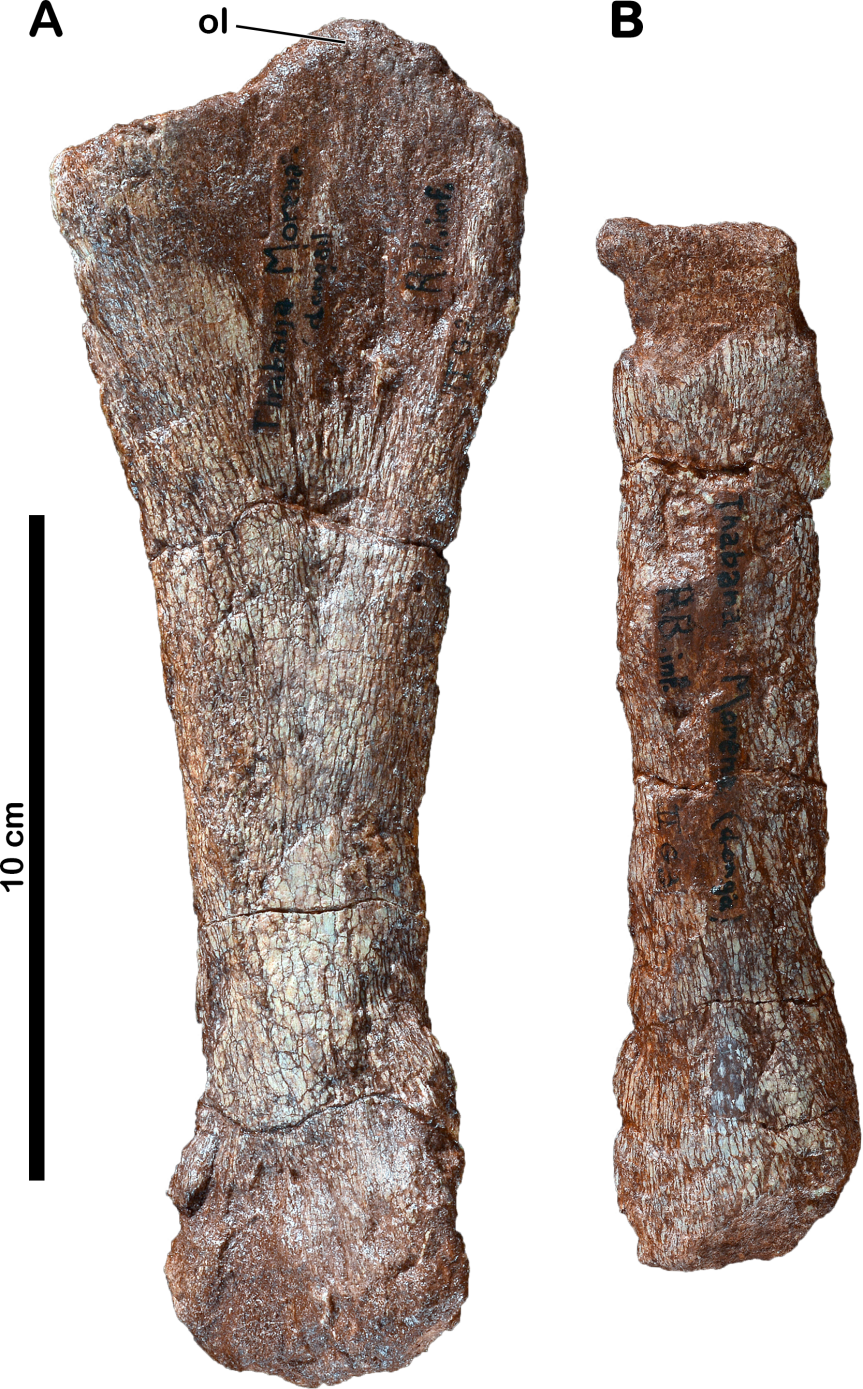
Forelimb bones of *Meroktenos*. (A) Left ulna, MNHN.F.LES351b, in medial view. (B) Left? radius, MNHN.F.LES351c. ol, olecranon. (Photo credit: L Cazes.)

**Referred material.** MNHN.F.LES351. Material associated with the holotype, previously illustrated by Costedoat (1962): cervical vertebra (MNHN.F.LES351a; Fig. 2); left ulna (MNHN.F.LES351b; Fig. 3); left? radius (MNHN.F.LES351c; Fig. 3). This material was originally catalogued under a different field number. Costedoat (1962:58) wrote regarding MNHN.F.LES16 and MNHN.F.LES351 : “Seven red elements of earthy appearance, found together in this site, certainly represent the same individual.” However, in absence of proof that the holotype and the referred material belong to the same animal, and presently unable to explain the two different field numbers, we only tentatively refer MNHN.F.LES351 to *Meroktenos* gen. nov.

**Revised diagnosis** A basal sauropodomorph with the following unique combination of characters: depth of the iliac blade (from the most dorsal point of the supracetabular crest to the dorsal margin of the ilium) being 60% of the total height of the ilium (all other basal sauropodomorphs from Southern Africa have a ratio <0.5), subtriangular postacetabular process, very stocky femur (robustness index: 2.09, with the exception of *Antetonitrus* all other basal sauropodomorphs from Southern Africa have an index >2.18) with a straight shaft in anterior and lateral views, shaft significantly wider lateromedially than anteroposteriorly deep (eccentricity: 1.58, except *Antetonitrus* all the other basal sauropodomorphs from Southern Africa have an index <1.41) and bearing an oblique fourth trochanter.

**Associated fauna** Ellenberger (1970) reported *Gryponyx sp*. and crocodilian material, as well as tridactyl tracks from the upper Elliot Formation of Thabana Morena. Unfortunately, no more details about these latter specimens were given and, except for one vertebra and a cast of a humerus, the whereabouts of the other fossils is currently unknown. More recently, during field trips in 2001 and 2008, other fossil remains including tritheledontid, tritylodontid and *Massospondylus sp.* material were found in the upper Elliot Formation near Thabana Morena (B Battail, pers. comm., 2014). They are housed in the MNHN collections.

**Comments** The material we describe here was first illustrated and briefly described by Costedoat (1962). She referred it to a ‘prosauropod’ dinosaur, specifically to the genus *Gryponyx*, but expressed reservations regarding this identification. Later, it was referred to an already existing genus: *Melanorosaurus* by Gauffre, who created a new Early Jurassic species of basal sauropodomorph: *M. thabanensis* (Gauffre, 1993a). Subsequently, Gauffre (1996), in his PhD thesis synonymized *M. thabanensis* with a new Late Triassic species “*Kholumolumosaurus ellenbergerorum*” (i.e., ‘The Maphutseng Dinosaur’) (Gauffre, 1996:38). This latter species has never been formally published, and must be treated as a nomen nudum. Thus, aside from the fact that both taxa are anatomically different (see below), their synonymy is not valid.

## Description

### Cervical vertebra

The only cervical vertebra preserved (MNHN.F.LES351a) is part of the referred material (Fig. 2). The preserved part of the centrum is 87 mm long and the vertebra is 60 mm high. Unfortunately, these values are not very informative given that the vertebra is badly crushed and transversely compressed. The diapophyses are not visible. The distal ends of both prezygapophyses and of the left postzygapophysis are broken. The right side of the vertebra is better preserved than the left one. The neural spine is short and low, its anteroposterior length is 33% of the total centrum length. Based on this ratio and on the overall shape of the vertebra, the latter is probably an anterior element of the cervical series. As in *Adeopapposaurus* (Martínez, 2009), the centrum is low and it seems that the postzygapophyses did not extend beyond its posterior margin. In right lateral view, the parapophysis is eroded but still distinct on the anteroventral surface of the centrum. The vertebra is too badly preserved to infer the presence or absence of a ventral keel.

### Dorsal? neural arch

**Figure 4:**
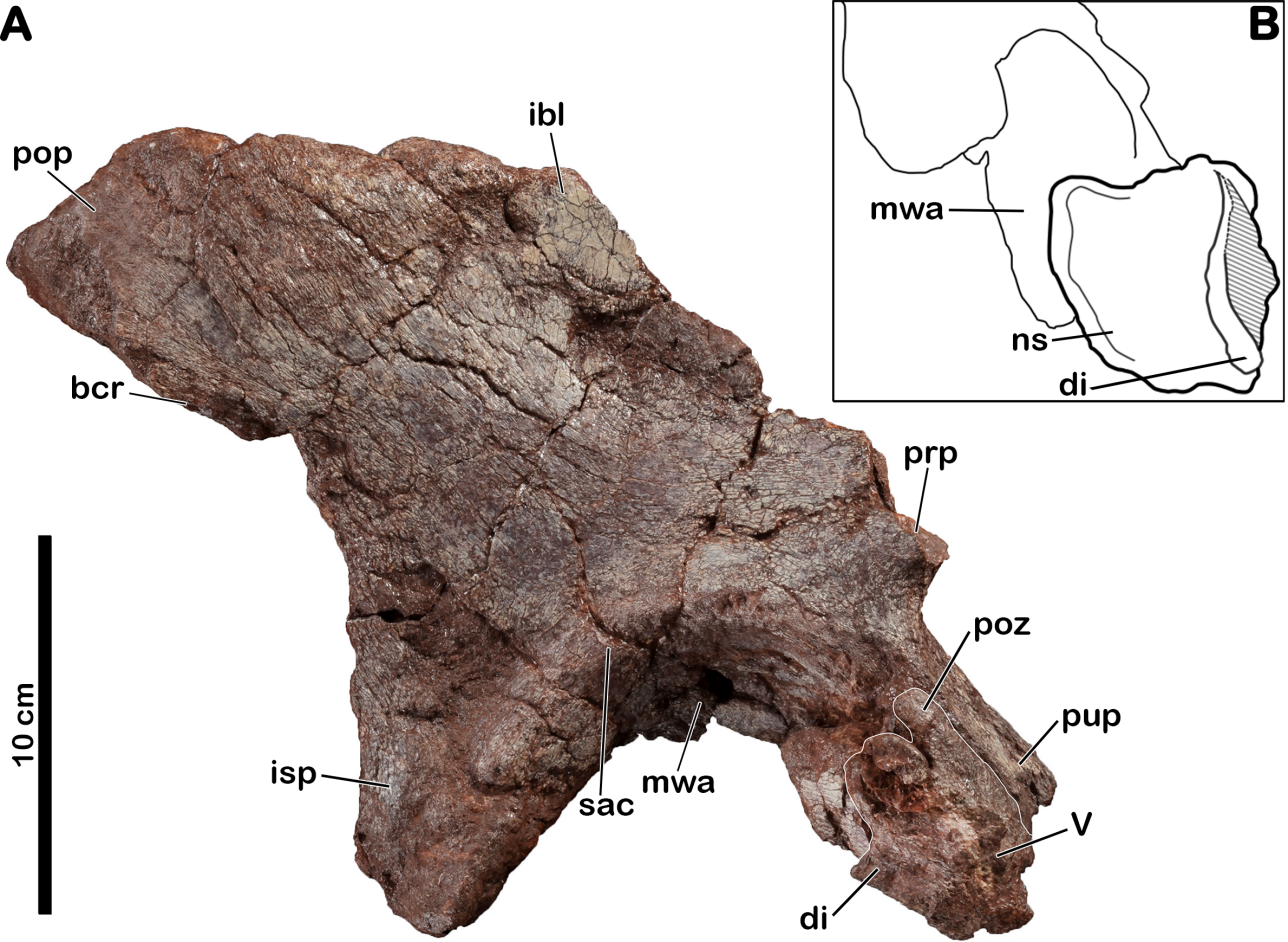
Right ilium of *Meroktenos*, MNHN.F.LES16a. (A) Lateral view. (B) Interpretative drawing of a close-up of the acetabulum in ventral view, showing the neural arch of the vertebra. bcr, brevis crest; di, diapophysis; ibl, iliac blade; isp, ischial peduncle; mwa, medial wall of the acetabulum; ns, neural spine; prp, preacetabular process; pop, postacetabular process; poz, postzygapophysis; pup, pubic peduncle; sac, supracetabular crest; V, vertebra. (Photo credit: L Cazes.)

An isolated neural arch of vertebra is preserved in contact with the pubic peduncle of the right ilium (MNHN.F.LES16a) of *Meroktenos* (Fig. 4). The neural spine is 58 mm long at its base. It is adhered to the acetabular region of the pubic peduncle, with the dorsal part of the neural spine pointing toward the medial margin of the acetabulum. The anterior part of the vertebra is located toward the distal end of the pubic peduncle. The neural spine of the vertebra is stout, anteroposteriorly elongated (relative to the orientation of the vertebra) and dorsoventrally low. The eroded postzygapophyses, separated by an interpostzygapophyseal notch, raise at the base of the neural spine. In lateral view, a slight projecting posterodorsal corner is visible. The base of the left diapophysis, anteroposteriorly developed, protrudes laterally.

With respect to the shape and the length of the neural arch, it is likely that it comes from a dorsal vertebra, probably a posterior one. Considering the overall shape, the neural arch of *Meroktenos* is very stout and resembles dorsal vertebrae of *Plateosaurus* (Moser, 2003). In comparison with *Melanorosaurus readi* anterior caudal vertebrae that exhibit tall neural spines (Galton, Van Heerden & Yates, 2005; NM QR3314), it seems unlikely that this neural arch is from a caudal vertebra.

### Left ulna

A left ulna (MNHN.F.LES351b) was found amongst referred assemblage (Fig. 3). It is 203 mm long on its medial side, which is better preserved than the lateral one, the latter being badly crushed and eroded. The development of the anteromedial and anterolateral processes is not visible, the proximal and distal ends of the bone being very damaged. Nonetheless, the well-developed olecranon process is visible proximomedially and resembles the olecranon process of the ulna of *Melanorosaurus* (Bonnan & Yates, 2007) in terms of proportions. The radial fossa is shallow but visible. At mid-shaft, the anteroposterior width of the ulna is 42 mm. The distal end is slightly more expanded than the shaft anteroposteriorly.

### Left? radius

Only the shaft of a radius (MNHN.F.LES351c), which is part of the referred material, is preserved (Fig. 3B). At mid-shaft, the anteroposterior width of the bone is 30 mm. The proximal and distal ends are missing and the outer surface of the bone is highly eroded, rendering any orientation impossible. We assume that this element is probably a left one, as it was found in close association with the left ulna (Fig. 3A).

### Right ilium

The right ilium (MNHN.F.LES16a) of *Meroktenos* is preserved, although not entirely (Fig. 4). Most of the preacetabular process, the distal end of the pubic peduncle and the anterodorsal part of the ischial blade are missing. The posteroventral corner of the postacetabular process is slightly eroded. An isolated neural arch of a vertebra is preserved against the pubic peduncle (see above).

The dorsal margin of the ilium is relatively straight posteriorly. The iliac blade appears to retain the condition observed in most non-sauropod sauropodomorphs, which consists of an anteroposterior elongation and a dorsoventral reduction. These two conditions are modified in Sauropoda, which possess a strongly convex dorsal margin (Gauthier, 1986) and a high iliac blade (McIntosh, 1990). Mediolaterally, the iliac blade is thinner dorsal to the acetabulum than at the level of the postacetabular process, forming a concave area on the lateral surface on the bone that extends ventrally to a point close to the acetabular margin. In contrast, this surface is restricted to the dorsal half of the iliac blade in some non-sauropod sauropodomorphs, such as *Lufengosaurus* (Young, 1941), *Plateosaurus* (Moser, 2003) and *Riojasaurus* (Bonaparte, 1971). Above the acetabulum, the iliac blade is very high being approximately two-thirds of the total height of the ilium (Table 4). As in most sauropodomorphs, there is no marked brevis crest on the ilium of *Meroktenos* and thus, the brevis fossa is lacking.

The postacetabular process is subtriangular, with oblique dorsal and ventral margins converging at the most distal point. This condition is in marked distinction to the subrectangular profile observed in most basal sauropodomorphs such as *Efraasia* (Galton, 1973), *Jingshanosaurus* (Zhang & Yang, 1994), *Lessemsaurus* (Pol & Powell, 2007), *Melanorosaurus* (Galton, Van Heerden & Yates, 2005), *Thecodontosaurus* (Benton et al., 2000) or *Yunnanosaurus youngi* (Lü et al., 2007). The postacetabular process is elongated anteroposteriorly, as in most basal sauropodomorphs.

The acetabular region is dorsoventrally low and seems to be transitional, in terms of anteroposterior extension, between long acetabula like those of *Anchisaurus* (Galton & Cluver, 1976) and *Y. youngi* (Lü et al., 2007), and narrower acetabular regions like in *Lessemsaurus* (Pol & Powell, 2007) or *Riojasaurus* (Bonaparte, 1971). The supracetabular crest is eroded, thus it is not possible to say whether it was laterally expanded or not. However, the anteroposterior extension is visible. Posteriorly, the crest rises slowly from the base of the ischial peduncle to form a slight ridge in the posterodorsal region of the acetabulum. The supracetabular crest follows the curvature of the acetabulum until at least the base of the pubic peduncle. The neural arch of the vertebra obscures the position where the crest merges into the pubic peduncle. As in all sauropodomorphs, the acetabulum is completely perforate. The acetabular surface of the pubic peduncle is twice as wide as the corresponding acetabular surface of the ischial peduncle. The medial wall extends along the dorsal margin of the acetabulum and slightly onto the posterior margin along the ischial peduncle. The anterior and dorsal margins of the acetabular surface are concave, whereas it is slightly convex on its posterior margin.

The pubic peduncle extends anteroventrally for approximately 600 mm, before terminating at an incomplete distal articular end. Its anterior surface is slightly concave. The pubic peduncle has a triangular cross-section in anteroventral view.

The ischial peduncle extends ventrally and very slightly posteriorly approximately 800 mm. The well-developed ischial peduncle is consistent with the condition in all non-sauropod sauropodomorphs. In contrast, this process becomes highly reduced in Sauropoda (Upchurch, Barrett & Dodson, 2004). In *Meroktenos*, the ischial peduncle appears to be longer than the pubic peduncle, probably due to its more slightly vertical orientation and to the broken extremity of the pubic peduncle. The proportions of the two peduncles are often similar in non-sauropod sauropodomorphs, except in *Y. youngi* (Lü et al., 2007), where the pubic peduncle is significantly longer. The ischial peduncle is subtriangular in lateral view, and in transverse cross-section (the base of the triangle is located anteriorly). It is not possible to assess with certainty if *Meroktenos* displays a heel on the posteroventral edge of the ischial peduncle, its extremity being slightly crushed and eroded. A peduncle heel is observed in *Plateosaurus* (Moser, 2003), *Riojasaurus* (Bonaparte, 1971), *Plateosauravus* (Van Heerden, 1979) and, to a lesser extent, in *Melanorosaurus* (Galton, Van Heerden & Yates, 2005).

**Figure 5:**
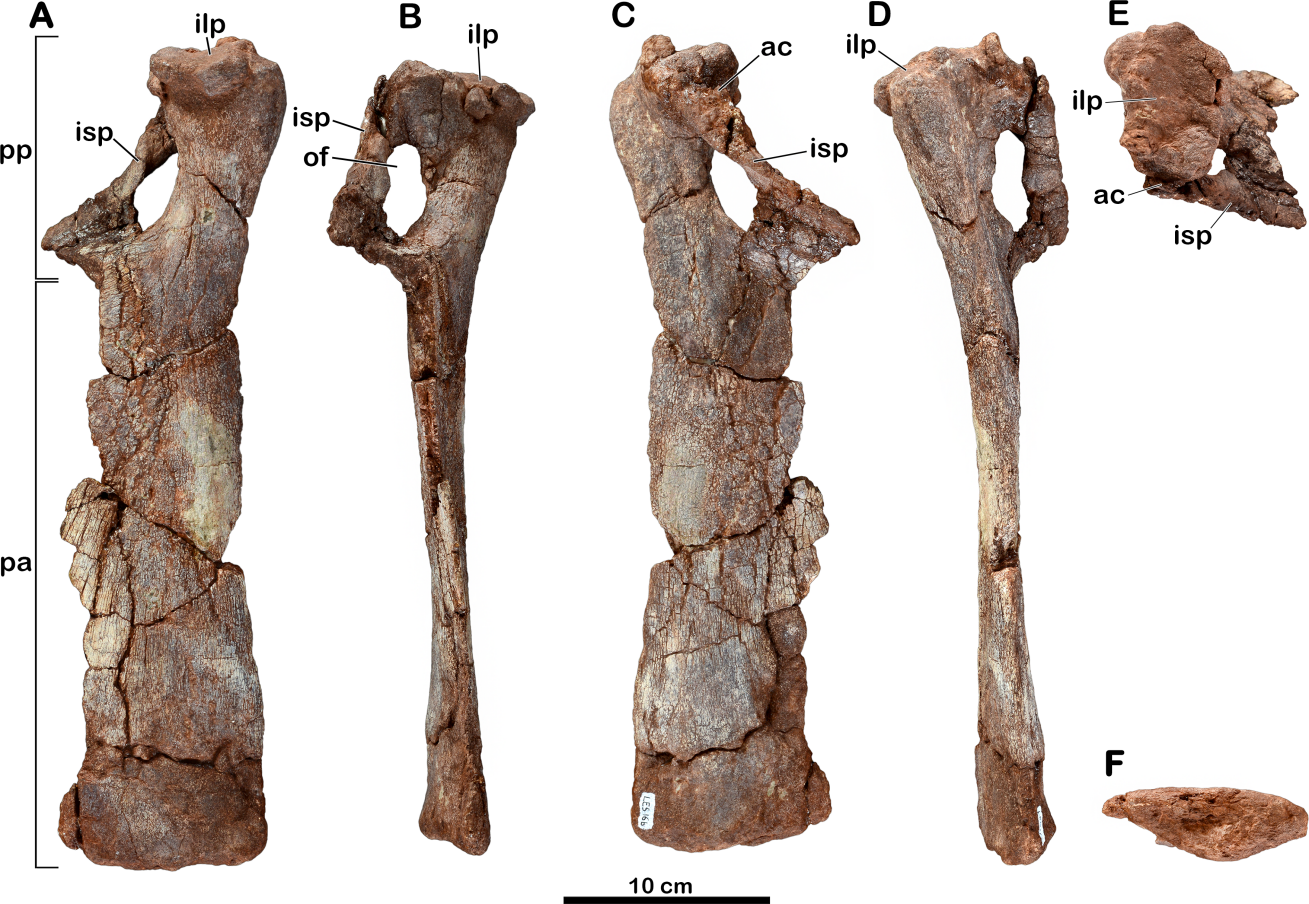
Left pubis of *Meroktenos*, MNHN.F.LES16b. (A) Anterior, (B) medial, (C) posterior, (D) lateral, (E) proximal, and (F) distal views. ac, acetabulum; ilp, iliac peduncle; isp, ischial peduncle; of, obturator foramen; pa, pubic apron; pp, pubic plate. (Photo credit: L Cazes.)

### Left pubis

The left pubis (MNHN.F.LES16b) of *Meroktenos* is mostly complete, with only the medial margin of the pubic apron not entirely preserved (Fig. 5). Its length is approximately 400 mm and the maximum transversal width on the pubic apron is 90 mm. As in most basal sauropodomorphs, the pubis is long and slender relative to its mediolateral expansion. Conversely, Sauropoda have more robust and broader pubes. The maximum mediolateral width of the pubis (measured along the obturator plate) represents approximately 30% of the total proximodistal length (this value is between 30–35% in other basal sauropodomorphs). In anterior view, the lateral margin of the pubis presents a concave outline, which is more or less marked depending on the genus (as in *Y. youngi*, Lü et al., 2007 or *Plateosaurus*, Moser, 2003, respectively). The iliac peduncle is relatively flat in medial view, with the peduncle being suboval in outline in proximal view. The long axis of the peduncle is oriented anterolaterally to posteromedially. Posteromedially, the acetabular portion of the pubis is not clearly defined, although it is necessarily situated between the iliac and ischiac peduncles. The ischiac peduncle is a slender blade, it tapers anteroventrally to a few millimeters wide in medial view. The thin part represents the majority of the length of the peduncle. The obturator foramen extends proximodistally and is suboval and relatively large, contrasting with the subcircular and reduced foramina of sauropod pubes (Upchurch, Barrett & Dodson, 2004). The overall proportions of the foramen are close to the condition observed in *Adeopapposaurus* (Martínez, 2009). The pubic plate is short, occupying approximately one-quarter of the total length of the pubis. The same condition is known in *Plateosaurus* (Moser, 2003), *Adeopapposaurus* (Martínez, 2009) and *Lufengosaurus* (Young, 1941). It differs from more extensive plates, like those of *Lessemsaurus* (Pol & Powell, 2007) or *Vulcanodon* (Raath, 1972).

Distal to the pubic plate, the pubis is flat and lateromedially expanded, forming the pubic apron. The latter occupies approximately three-quarters of the entire pubic length. The lateral margin of the apron is nearly straight in anterior view, as in *Melanorosaurus* (Galton, Van Heerden & Yates, 2005) and *Plateosaurus* (Moser, 2003), and is dorsoventrally thicker than the medial margin. The medial edge, which forms the pubic symphysis, is thin and not fully preserved.

The distal end of the pubis has a maximum lateromedial width that is approximately 34% of the pubis length. This is very close to the condition of *Adeopapposaurus* (Martínez, 2009) or *Lessemsaurus* (Pol & Powell, 2007) and less than the 44% and 50% measured for *Antetonitrus* (McPhee et al., 2014) and *Vulcanodon* (Raath, 1972), respectively. The distal end is not markedly anteroposteriorly expanded, as in some other basal sauropodomorphs, such as *Coloradisaurus* (Apaldetti, Pol & Yates, 2013) and *Plateosaurus* (Moser, 2003). Instead, the condition in *Meroktenos* is closer to that in *Melanorosaurus* (Galton, Van Heerden & Yates, 2005) or *Riojasaurus* (Bonaparte, 1971). The distal surface is subtriangular with a straight anterior edge, whereas the posterior edge is convex.

### Right femur

**Figure 6:**
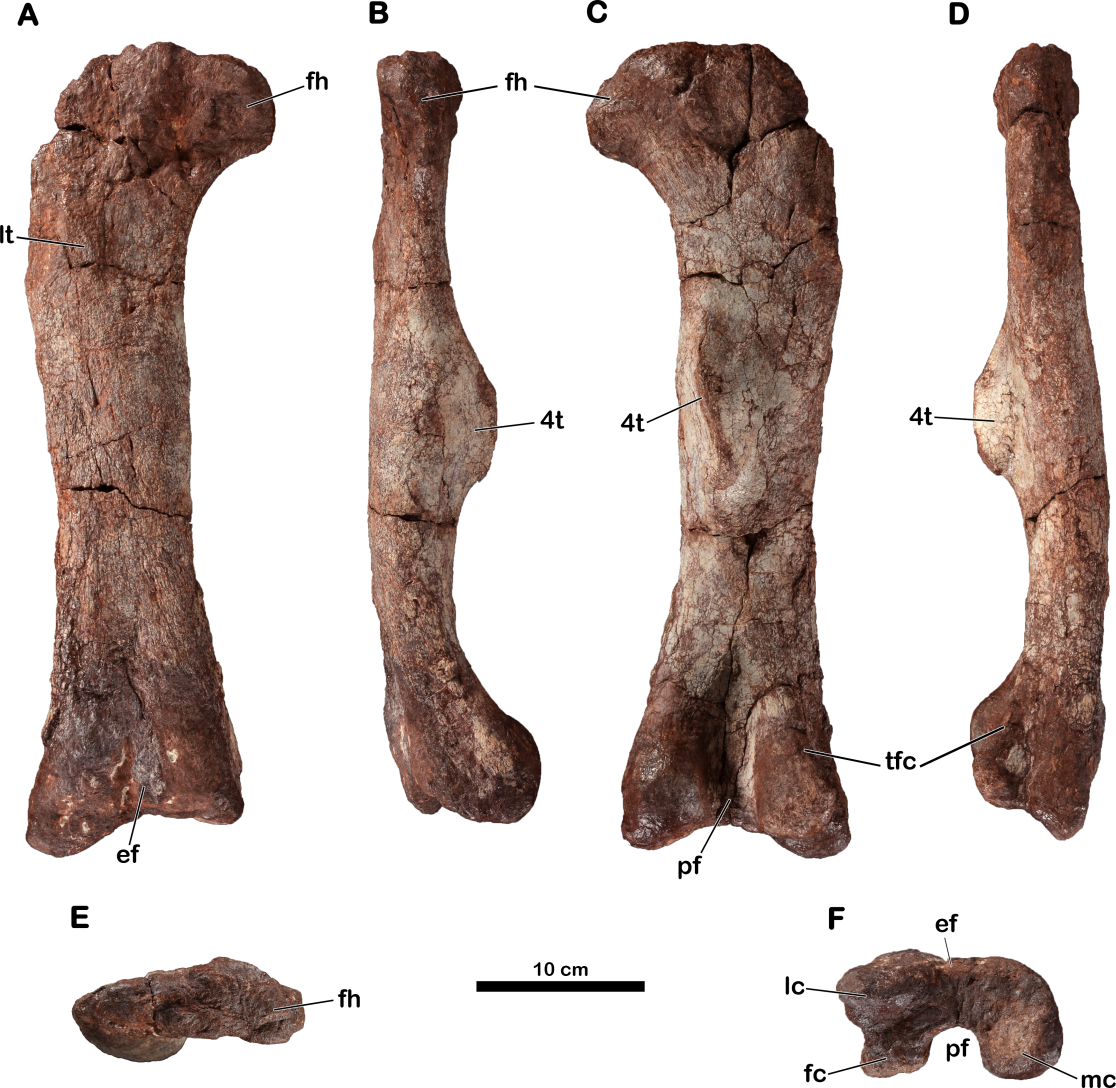
Right femur of *Meroktenos*, MNHN.F.LES16c. (A) Anterior, (B) medial, (C) posterior, (D) lateral, (E) proximal, and (F) distal views. ef, extensor fossa; fc, fibular condyle; fh, femoral head; lc, lateral condyle; lt, lesser trochanter; mc, medial condyle; pf, popliteal fossa; tfc, tibiofibular crest; 4t, fourth trochanter. (Photo credit: L Cazes.)

The holotypic right femur (MNHN.F.LES16c) is 480 mm long (Table 2) and is generally well-preserved (Fig. 6). Its precise taphonomic circumstances are not known, but we assume that it was found lying on its posterior surface because the anterior surface is eroded (probably because of weathering). The proximal and distal ends do not seem to have undergone distortion; only the proximolateral corner of the femur is broken.

**Table 2:** Comparative measurements of the femora of *Meroktenos* and several Upper Triassic and Lower Jurassic basal sauropodomorph specimens from Southern Africa (sorted by length).

Specimens	Total length	Mediolateral width femoral head	Anteroposterior width femoral head	Midshaft mediolateral width	Midshaft anteroposterior width
*Massospondylus* SAM-PK-402	247	72	30	32	27
*Massospondylus* SAM-PK-391	250	–	14,5	28	24
*Massospondylus* SAM-PK-397	359	94	39	42	46
*Massospondylus* SAM-PK-393	390	87	51	43	51
*Massospondylus* MNHN.F.LES15-7	415	85	54	50	59
***Meroktenos* MNHN.F.LES16c**	**480**	**153**	**57**	**82**	**52**
*Gryponyx* SAM-PK-7919	535	–	44	67	68
*Melanorosaurus* NM QR1551	623	139	80	93	66
*Melanorosaurus* SAM-PK-3450	624	173	69	103	77
*Aardonyx* BP/1/6510	682	188	–	91	90
*Euskelosaurus* SAM-PK-330	700	195	76	90	97
*Antetonitrus* BP/1/4952	775	208	114	142	94
‘Maphutseng dinosaur’ MNHN.F.LES394	780	220	110	110	95

Specimens	Distal mediolateral width	Distal anteroposterior width	Circumference under 4th trochanter	Distal expansion 4th trochanter
*Massospondylus* SAM-PK-402	–	–	96	125
*Massospondylus* SAM-PK-391	57	43	90	112
*Massospondylus* SAM-PK-397	101	69	149	180
*Massospondylus* SAM-PK-393	98	70	141	183
*Massospondylus* MNHN.F.LES15-7	91	75	175	198
***Meroktenos* MNHN.F.LES16c**	**136**	**78**	**230**	**280**
*Gryponyx* SAM-PK-7919	107	121	205	290
*Melanorosaurus* NM QR1551	183	88	266	305
*Melanorosaurus* SAM-PK-3450	172	110	273	350
*Aardonyx* BP/1/6510	169	110	284	380
*Euskelosaurus* SAM-PK-330	177	118	298	390
*Antetonitrus* BP/1/4952	270 (deformed)	150	410	450
‘Maphutseng dinosaur’ MNHN.F.LES394	195	130	320	425

The outline of the proximal surface is mediolaterally elongated (the maximum transverse length is approximately three times the maximum anteroposterior length) and is suboval. The femoral head is slightly directed anteriorly. In anterior and posterior views, the femoral head is roughly rectangular, extending perpendicularly from the long axis of the shaft. Its anteroposterior length is inferior to its proximodistal height. In anterior and posterior views, the femoral shaft is straight (i.e., following the shaft axis established as the line perpendicular to the distal transversal plan by Gauffre, 1993a). In lateral and medial views, the shaft is also straight, lacking a sigmoid curvature: this is also observed in *Antetonitrus* (McPhee et al., 2014), *Camelotia* (Galton, 1985) and *Melanorosaurus* (Van Heerden & Galton, 1997), as well as in Sauropoda (Upchurch, Barrett & Dodson, 2004).

The femur is relatively short and stout. Its robustness index (total length/circumference: a robustness index less than or equal to 2 indicates a really stout femur) is 2.09 (Table 3), whereas other forms such as *Aardonyx* (Yates et al., 2010) or *Massospondylus* (MNHN.F.LES15-7) have indices of 2.40 and 2.37, respectively. However, it is not as robust as in *Antetonitrus* (McPhee et al., 2014), which exhibits a robustness index of 1.89 (Table 3). The shaft is anteroposteriorly compressed: its eccentricity ratio (mediolateral width at midshaft/anteroposterior width at midshaft: a ratio close to 1 indicates a subcircular shaft) being 1.58, whereas *Aardonyx* (Yates et al., 2010) and *Melanorosaurus* (SAM-PK-3450) have ratios of 1.01 and 1.34, respectively. This compression is also observed in *Antetonitrus* (eccentricity: 1.51) (Table 3). In comparison, the majority of other basal sauropodomorphs present a subcircular cross-section of the mid-shaft of the femur.

**Table 3:** Comparative ratios of the femora. Comparative ratios measured on the femora of *Meroktenos* and 23 other sauropodomorph specimens (sorted by ascending eccentricity). Eccentricity, mediolateral width at midshaft/anteroposterior width at midshaft; Robustness index, total length/circumference.

Specimens	Eccentricity	Robustness index
*Massospondylus* SAM-PK-393	0.84	2.77
*Massospondylus* MNHN.F.LES15-7	0.85	2.37
*Plateosaurus* MB.R.4404.62	0.87	2.35
*Massospondylus* SAM-PK-397	0.91	2.41
*Euskelosaurus* SAM-PK-330	0.93	2.35
*Aardonyx* BP/1/6510	0.96	2.4
*Gryponyx* SAM-PK-7919	0.99	2.61
*Ruehleia* MB.R.4718.99	1.02	2.81
‘Maphutseng dinosaur’ MNHN.F.LES394	1.16	2.44
*Melanorosaurus* NM QR3314	1.16	2.25
*Massospondylus* SAM-PK-391	1.17	2.78
*Massospondylus* SAM-PK-402	1.19	2.6
*Melanorosaurus* SAM-PK-3450	1.34	2.29
*Eucnemesaurus* BP/1/6234	1.34	2.33
*Melanorosaurus* NM QR1551	1.41	2.34
*Lapparentosaurus* MNHN.F.MAA67	1.43	2.47
*Antetonitrus* BP/1/4952	1.51	1.89
*Tazoudasaurus* CPSGM To1-105	1.54	2.25
***Meroktenos* MNHN.F.LES16c**	**1.58**	**2.09**
*Diplodocus* CM.84	1.6	2.55
*Camarasaurus* YPM mount	1.79	?
*Diplodocus* CM.94	1.82	?
*Nigersaurus* MNHN.F.GDF327	1.92	2.45
*Cetiosaurus* OUMNH J13615	1.93	2.36

The lesser trochanter appears as a low, elevated scar upon the anterior femoral surface rather than as a raised process. However, the anterior surface is much too eroded to assert with certainty the full development of the lesser trochanter as well as its orientation relative to the long axis of the femoral shaft. The lesser trochanter is well removed from the lateral edge and is thus not visible in posterior view.

The fourth trochanter is located on the posterior face, at the mid-length of the femur. Whereas the trochanter is relatively proximal in basal saurischians like *Saturnalia* (Langer, 2003), it is more distally situated in most other basal sauropodomorphs (Rauhut et al., 2011). In *Meroktenos*, the fourth trochanter straddles the midpoint of the femur, as in *Aardonyx* (Yates et al., 2010). On the transverse axis, the fourth trochanter is close to the medial margin of the femur. Gauffre (1993a) suggested a close relationship between *Melanorosaurus* and *Meroktenos* based primarily on this character. In *Melanorosaurus* (SAM-PK-3450 and NM QR1551), like in most basal sauropodomorph taxa, the degree of projection of the fourth trochanter is well-developed. In *Meroktenos*, the apical surface of the fourth trochanter is a little damaged and the distal part is broken but was probably slightly pendant. In posterior view, the fourth trochanter is slightly sigmoid. In cross section, it is triangular. It has an oblique orientation (based on the orientation of the main axis of the fourth trochanter relative to the long axis of the shaft), a character which was highlighted by Gauffre (1993a) and which helped, in his opinion, to distinguish *Meroktenos* from *Melanorosaurus*. Distally, the fourth trochanter rises steeply from the shaft but merges gradually with the shaft proximally. In lateral view, the fourth trochanter has the profile of an asymmetrical trapezoid, with the proximal edge sloping upwards to merge with the shaft, whereas the distal contact with the shaft is relatively steep. This shape is intermediate between what is observed in *Antetonitrus* (McPhee et al., 2014) and what was described for *Riojasaurus* (Bonaparte, 1971). The medial surface of the fourth trochanter lacks an adjacent fossa for the insertion of the muscle *caudofemoralis longus* and merges directly into the medial edge of the shaft.

The distal end of the femur is not distorted, as it is sometimes the case for other specimens (McPhee et al., 2014; MNHN.F.LES15-7). The distal condyles are subspherical and do not seem to be anteroposteriorly compressed. The medial condyle is subequal in size to the fibular and lateral condyles together (Fig. 6F). The mediolateral expansion of the distal end relative to the transverse width of the shaft is not as pronounced as in *Lessemsaurus* (Pol & Powell, 2007) or *Plateosaurus* (Moser, 2003). Anteriorly, the extensor fossa is visible but shallow. Posteriorly, the popliteal fossa is well-defined. The tibiofibular crest is neither sharp nor elongated as is the case in *Plateosaurus* (Moser, 2003). However, the crest is not as reduced as in *Antetonitrus* (McPhee et al., 2014), in which the condition is closer to more derived taxa such as *Tazoudasaurus* (Allain & Aquesbi, 2008) or *Shunosaurus* (Zhang, 1988).

### Right metatarsal II

**Figure 7:**
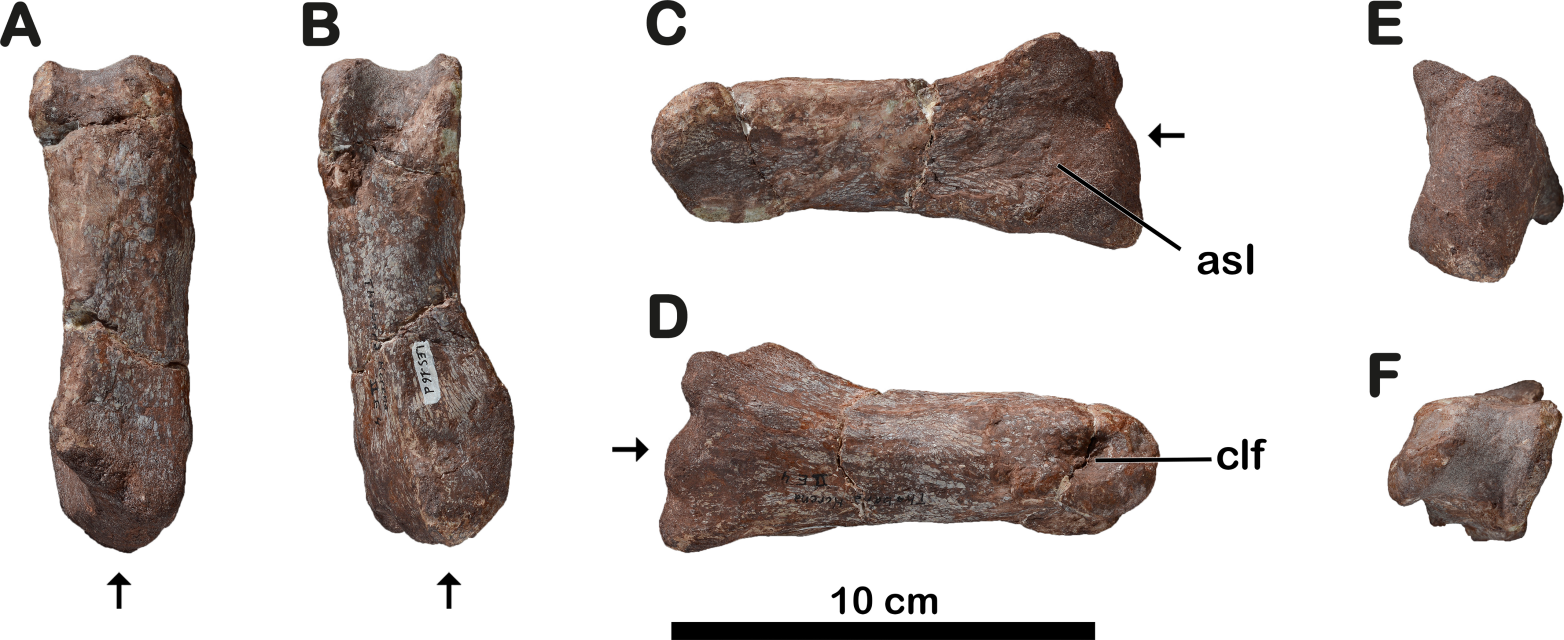
Right metatarsal II of *Meroktenos*, MNHN.F.LES16d. (A) Dorsal, (B) ventral, (C) medial, (D) lateral, (E) proximal, and (F) distal views. asI, articular surface for metatarsal I; clf, collateral ligament fossa. Arrows indicate the proximal end of the bone. In (E) and (F), the lateral side of the bone is at the right side of the picture. (Photo credit: L Cazes.)

A right metatarsal II (MNHN.F.LES16d) is amongst the material pertaining to *Meroktenos* (Fig. 7). It is slender and exhibits lateromedially narrow extremities, although the latter are eroded and were probably more expanded.

The shaft of the metatarsal II is long and straight. The bone is 116 mm long, and its minimal width is 36 mm. As in other basal sauropodomorphs, the minimum transverse width is approximately 31% of the proximodistal length of the bone in *Meroktenos*. Conversely, more derived sauropodomorphs like *Antetonitrus* (McPhee et al., 2014) or *Tazoudasaurus* (Allain & Aquesbi, 2008) exhibit a more robust pes morphology (the minimum transverse width being approximately 50% of the proximodistal length of the metatarsal). In proximal view, the articular surface is subrectangular, asymmetrical and more elongated dorsoventrally than expanded mediolaterally. It is not biconcave as in most basal sauropodomorphs, although this could be a result of erosion. The medial edge is concave and extends ventrally into a ventromedial flange, however the lateral margin is slightly convex. The ventromedial flange is not very developed (maybe as a result of erosion), in comparison to derived sauropodomorphs like *Antetonitrus* (McPhee et al., 2014) or *Tazoudasaurus* (Allain & Aquesbi, 2008). The distal condyles are eroded and were probably more expanded tranversely. A deep collateral fossa is visible on the lateral surface of the lateral condyle whereas the medial condyle only exhibits a shallow fossa.

## Comparisons

Among basal sauropodomorphs, ten genera other than *Meroktenos* are known from the Late Triassic of southwestern Gondwana (southern Africa and South America). Of these, *Euskelosaurus*, currently considered a nomen dubium by most authors (Yates, 2004), is not included in the following comparisons. *Blikanasaurus* (Galton & Van Heerden, 1998) and *Unaysaurus* (Leal et al., 2004) are not consulted further because of the absence of overlapping elements with those known from *Meroktenos*.

**Figure 8:**
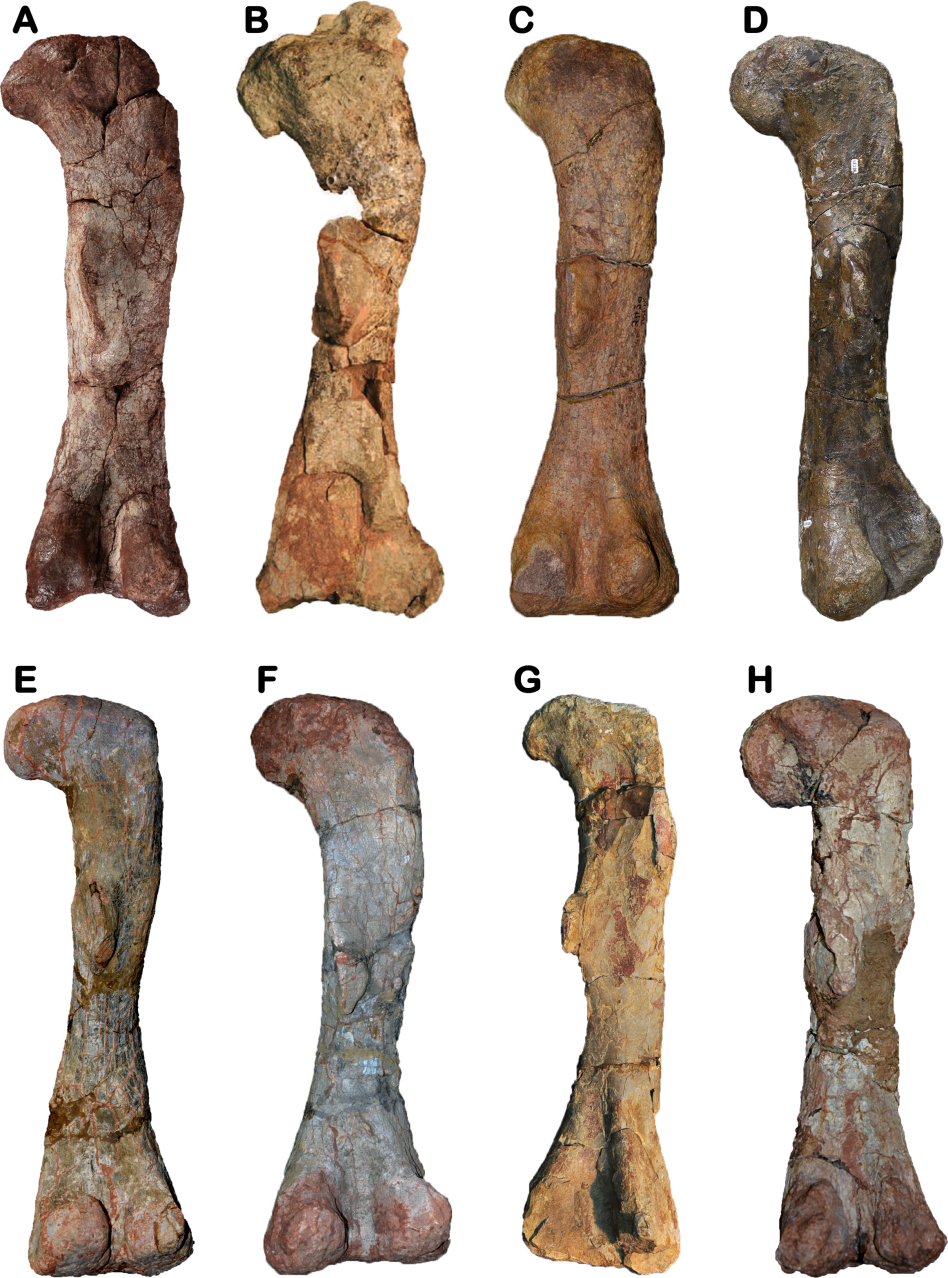
Comparative figure showing the posterior view of the femora of Late Triassic basal sauropodomorphs from Southwestern Gondwana. (A) Right femur of *Meroktenos thabanensis* MNHN.F.LES16c (photo credit: L Cazes). (B) Right femur of *Eucnemesaurus entaxonis* BP/1/6234 (modified from McPhee et al., 2015). (C) Left femur of *Melanorosaurus* readi SAM-PK-3450 (photo credit: C. Peyre de Fabrègues). (D) Right femur of ‘The Maphutseng dinosaur’ MNHN.F.LES394 (photo credit: L Cazes). (E) Right femur of *Coloradisaurus brevis* PVL 5904 (photo credit: C Peyre de Fabrègues). (F) Right femur of *Lessemsaurus sauropoides* PVL 4822/65 (photo credit: C Peyre de Fabrègues). (G) Left femur of *Mussaurus patagonicus* MLP 68-II-27-1 specimen A (photo credit: A Otero). (H) Left femur of *Riojasaurus incertus* PVL 3808 (photo credit: C Peyre de Fabrègues). All specimens are presented at the same scale to emphasize the disparity of proportions.

### Comparison with Triassic basal sauropodomorphs from Southern Africa

Several femoral fragments of *Eucnemesaurus fortis* are known (Yates, 2007a) and, very recently, an articulated partial skeleton of a new species (*E. entaxonis*) was described (McPhee et al., 2015). *E. fortis* differs from *Meroktenos* regarding the eccentricity of the shaft of its femur, which is relatively subcircular, as in most basal sauropodomorphs. Another difference, also visible on the femur of *E. entaxonis*, concerns the fourth trochanter, which is distinctly more oblique in posterior view than in *Meroktenos* (Figs. 8A–8B). Furthermore, the trochanter is more strongly developed and more proximally located in lateral view in *Eucnemesaurus*. On the right ilium of *E. entaxonis*, the postacetabular process is subrectangular and is bordered by a deep brevis fossa, considered a potential autapomorphy of the species (McPhee et al., 2015). The femur of *E. entaxonis* is complete, so we were able to calculate its eccentricity and robustness index (Table 3). Unlike *E. fortis*, the cross section at midshaft of *E. entaxonis* is elliptical (eccentricity: 1.34), but still less than in *Meroktenos*. The femur is not particularly stout and has a robustness index greater than that in *Meroktenos* (2.33 versus 2.09, respectively).

Two femora are known for the genus *Plateosauravus* (SAM-PK-3602 and 3603) (Van Heerden, 1979). They are less stout than the femur of *Meroktenos*. On the specimen SAM-PK-3602, the fourth trochanter is complete. It projects far posteriorly and is subtriangular, thus differing from the subrectangular trochanter observed in *Meroktenos*.

The syntype series of *Melanorosaurus readi* originally included several elements of the pelvic girdle, including an ilium (Haughton, 1924:Fig. 44), which is currently lost. Based only on the illustration of Haughton (1924), we can note that the posterior margin of the postacetabular process of the ilium has a rectangular shape in lateral view in *Melanorosaurus*, whereas it is tapering in *Meroktenos*. Currently, the only material in common between the specimen from Thabana Morena and the syntype of *Melanorosaurus* is a broken proximal extremity of a pubis (SAM-PK-3449). Unfortunately, based only on the proximal extremity of the pubis, it is not possible to highlight marked differences. The femur originally catalogued under the accession number SAM-PK-3450 was found in a higher stratigraphic layer than the remaining type materials of *Melanorosaurus*. Despite its exclusion from the syntype series, and potentially from *M. readi* (see above), and pending a reexamination of the material, a cursory comparison of this element with the femur of *Meroktenos* follows. In *Melanorosaurus*, the head has a roughly hemispherical shape in anterior and posterior views. In *Meroktenos*, it is roughly rectangular. The lesser trochanter is close to the lateral margin of the bone in *Melanorosaurus*. In contrast, it is more medially located in *Meroktenos*. The profile of the fourth trochanter in medial view is nearly symmetrical in *Melanorosaurus*, whereas it is asymmetrical in *Meroktenos*. In posterior view, the fourth trochanter is completely straight proximodistally in *Melanorosaurus* (Fig. 8C). In *Meroktenos,* the fourth trochanter is oblique, with its distal end in a more lateral position than the proximal one (Gauffre 1993a). Finally, the femur SAM-PK-3450 is less stout, with a robustness index equal to 2.29 (in *Meroktenos*, 2.09), and less flattened, with an eccentricity ratio equal to 1.34 (in *Meroktenos*, 1.58) (Table 3).

The specimen referred to *Melanorosaurus readi* NM QR1551 includes a right ilium (NM QR1551/28), three pubes and a left femur (NM QR1551/51). With respect to the ilium, several features differ from the same element of *Meroktenos*. The postacetabular process of the ilium NM QR1551/28 is shallow dorsoventrally with a posteriorly directed subrectangular extremity. In *Meroktenos*, the end of the postacetabular process is triangular, and its dorsal and ventral margins are not parallel. In NM QR1551/28, the supracetabular crest is strongly developed, whereas it is less developed in *Meroktenos*. Finally, the iliac blade represents approximately half of the height of the bone in NM QR1551, in *Meroktenos* it is closer to two-thirds (Table 4). With respect to the pubes, the only visible difference is that the iliac peduncle is more than two times longer than wide in NM QR1551, but it is only slightly longer than wide in *Meroktenos*. The femur NM QR1551/51 differs from *Meroktenos* in general dimensions (Table 2) and by a more laterally located lesser trochanter visible in posterior view. The distal end of the fourth trochanter is situated in the proximal half of the femur in NM QR1551, whereas it lies beyond the distal half of the bone in *Meroktenos*. Finally, the femur NM QR1551/51 is more slender, has a robustness index equal to 2.34 (*Meroktenos* robustness index: 2.09), and exhibits a more subcircular shaft, with an eccentricity of 1.41 (Table 3).

**Table 4:** Comparative measurements of the ilia of *Meroktenos* and 9 other basal sauropodomorphs (sorted by ascending IB/TH ratio). IB/TH, dorsoventral height of the iliac blade/total height of the ilium.

Specimens	Total anteroposterior length (iliac blade)	IB/TH ratio
*Euskelosaurus* SAM-PK-3532	345	0.41
*Massospondylus* BP/1/4693	270	0.42
*Melanorosaurus* NM QR1551	400	0.43
*Ruehleia* MB.R.4718.101	510	0.44
‘Maphutseng dinosaur’ MNHN.F.LES375	620	0.49
*Massospondylus* BP/1/4934	270	0.5
*Riojasaurus* PVL 3808	?	0.52
*Lessemsaurus* PVL 4822/60	?	0.55
***Meroktenos* MNHN.F.LES16c**	**?**	**0.6**
*Plateosaurus* MB.R.4404.58	360	0.62

The second specimen referred to *Melanorosaurus readi* NM QR3314 is represented by an articulated skeleton. The ilia are in bad shape, making it impossible to compare with MNHN.F.LES16. The pubes, probably located under the sacrum, are not visible because the specimen is on exhibit. The femora are poorly preserved, and the distal part of the left femur is missing. The right femur is complete but damaged, so that the lesser and fourth trochanters are not visible. However, using the circumference and the length of the right femur (Table 2), we obtain a robustness index equal to 2.25, which is less robust than in *Meroktenos* (2.09). Because the femur is broken, the shape of the shaft can be easily observed. It is more circular than in *Meroktenos*, with an eccentricity equal to 1.16 (Table 3).

The ‘Maphutseng Dinosaur,’ much larger than *Meroktenos*, shows an appreciable disparity of general proportions with the material described here (Table 2). Regarding the ilium, the distal margin of the postacetabular process is rounded in the ilium from Maphutseng, whereas it is more pointed in *Meroktenos*. The ventral margin of the postacetabular process has a diagonal orientation in the material from Maphutseng, but *Meroktenos* exhibits a more horizontal orientation. The angle between the postacetabular process and the ischial peduncle is more acute in the Maphutseng material than in *Meroktenos*. The supracetabular crest is more dorsally located in the Maphutseng material, making the medial wall of the acetabulum more developed than in *M. thabanensis*. Finally, the ischial peduncle is lateromedially wider in the ilium from Maphutseng. On the pubis, the obturator plate is flattened anteriorly in the material of Maphutseng, whereas in *Meroktenos* it is more convex. Regarding the femur, the femoral head has a roughly hemispherical shape in anterior and posterior views for the Maphutseng material, and in *Meroktenos* the head is roughly rectangular. In the same views, the femur is sigmoid, but it is straight in *Meroktenos*. The distal margin of the fourth trochanter is convex, contrasting with the straight margin in *Meroktenos*. The position of the fourth trochanter along the mediolateral axis of the bone is close to central (Fig. 8D), but in *Meroktenos* it is completely medially located. Distally, the medial condyle is broader than the fibular and lateral condyles combined, whereas in *Meroktenos* they are subequal. Finally, the femur in the ‘Maphutseng Dinosaur’ is less robust (robustness index: 2.44) than *Meroktenos* (2.09) (Table 3). It is also more circular, with an eccentricity of 1.16 (Table 3).

### Comparison with Triassic basal sauropodomorphs from Southern America

Several postcranial elements of *Coloradisaurus* (Apaldetti, Pol & Yates, 2013) can be compared with elements from *Meroktenos*. The ischial process of the ilium is shorter and more subrectangular in *Coloradisaurus*. On the left pubis, the lateral margin of the pubic apron of *Coloradisaurus* is concave in anterior view, but it is nearly straight in *Meroktenos*. The cross section of the midshaft of the femur is clearly subcircular in *Coloradisaurus*. Furthermore, the fourth trochanter is located more proximally on the shaft in *Coloradisaurus* than in MNHN.F.LES16 (Fig. 8E). Finally, the main axis of the shaft of *Coloradisaurus* is sigmoid in lateral and medial views, whereas it is straight in *Meroktenos*.

An ilium, a pubis and two femora are known from *Lessemsaurus* (Pol & Powell, 2007). The pelvic elements are damaged. However, in *Lessemsaurus* the iliac blade of the ilium is less expanded relative to its total height than in *Meroktenos* (Table 4). On the right pubis, the lateral margin of the pubic apron of *Lessemsaurus* is concave in anterior view, whereas it is nearly straight in *Meroktenos*. Finally, the femur of *Lessemsaurus* is less stout (Fig. 8F) and more sigmoid in medial and lateral views than the femur of *Meroktenos*.

Pelvic elements as well as a femur are known from *Mussaurus* (Otero & Pol, 2013). The ilia are incomplete but the ventral part of the postacetabular process is preserved. It projects far beyond the ischial process and its ventral margin is horizontal, while it is oblique in *Meroktenos*. On the left pubis of *Mussaurus*, the distal end is more expanded anteroposteriorly than in MNHN.F.LES16. The fourth trochanter of the femur of *Mussaurus* is located more proximally on the shaft (Fig. 8G); it also projects more posteriorly and is less developed proximodistally than in *Meroktenos*.

In *Riojasaurus* (Bonaparte, 1971), the ischial peduncle exhibits a posteriorly projecting heel, which is absent in *Meroktenos*. Also, the ventral margin of the bone between the ischial peduncle and the postacetabular process is straight in lateral view in *Riojasaurus*, but it is concave in MNHN.F.LES16. Finally, the iliac blade above the acetabulum is low relative to the total height of the ilium in *Riojasaurus* (Table 4). The pubis of *Riojasaurus* is stouter and does not have the same shape in anterior view. The head of the femur of *Riojasaurus* is bulbous (Fig. 8H). The fourth trochanter is also more proximally located in *Riojasaurus* and presents a very steep ventral margin in medial view.

In conclusion, the material from Thabana Morena can no longer be referred to *Melanorosaurus* nor to any other basal sauropodomorph (Fig. 8). Following the principle of binomial nomenclature (article 5 of the ICZN, 1999), the scientific name of a species is a binomen. The type material of *Meroktenos thabanensis* being diagnostic (see above) and considering the two previous statements, the erection of a new genus is justified.

## Results

### Phylogenetic analysis

*Melanorosaurus* and *Meroktenos* have never been included together in a comprehensive cladistic analysis of basal sauropodomorphs, preventing a test of whether or not they are sister taxa. Here, we amend the data matrix from Apaldetti et al. (2014) to carry out a phylogenetic analysis. The matrix consists of 363 characters and 52 terminal taxa, including *Meroktenos*. The latter was scored based on the holotypic material (MNHN.F.LES16). An analysis also including scorings of the referred material was carried out, and led to the same consensus tree. Given the uncertainty surrounding the anatomy of *Gongxianosaurus*, this taxon was pruned from the data set. Despite the problems regarding its status (see above; Nair & Yates, 2014), the terminal unit *Melanorosaurus* includes both the type specimens (SAM-PK-3449 and SAM-PK-3450) and the referred specimens (SAM-PK-3532, NM QR1551 and NM QR3314).

**Figure 9:**
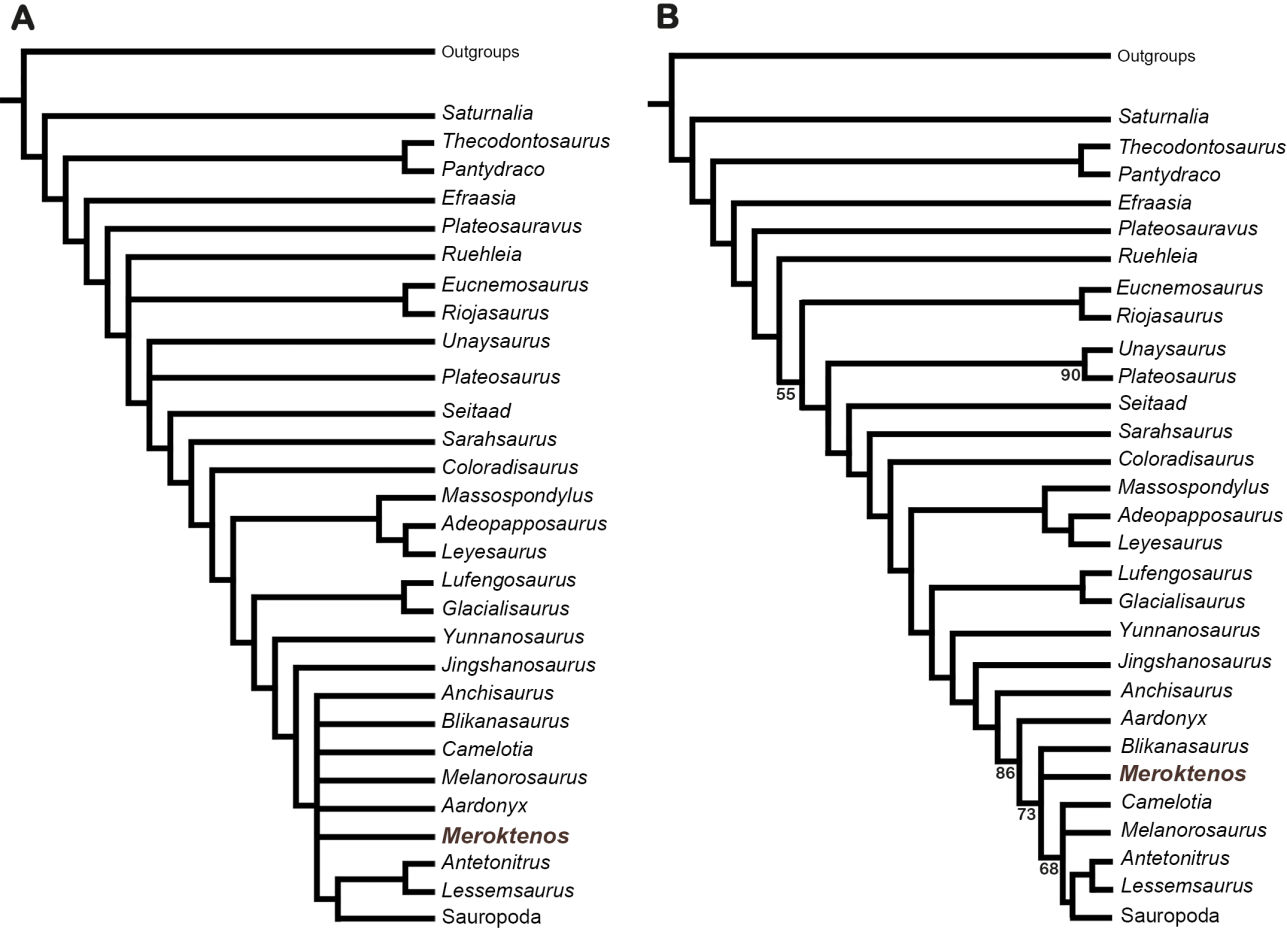
Phylogenetic position of *Meroktenos* based on the data set available from Apaldetti et al. (2014). (A) Strict consensus of the 440 most parsimonious trees (L = 1,308 steps). (B) Majority-rule consensus of the 440 most parsimonious trees. Sauropoda sensu Salgado, Coria & Calvo, (1997) (Peyre de Fabrègues, Allain & Barriel, 2015).

The matrix was analysed in PAUP 3.1 (Swofford, 1993) using a heuristic search with a random stepwise-addition of 100 replicates followed by TBR branch swapping. The analysis resulted in 440 most parsimonious trees (length = 1,262 steps, CI = 0.335, RI = 0.655). Based on this analysis, we produced a strict consensus tree (length = 1,308 steps; Fig. 9A) where *Antetonitrus* and *Lessemsaurus* form a small clade that is the sister group of Sauropoda (sensu Salgado, Coria & Calvo, 1997; Peyre de Fabrègues, Allain & Barriel, 2015). *Meroktenos* and *Melanorosaurus* are also recovered in the “apical” part of the tree (i.e., close to Sauropoda), within a polytomy including also *Anchisaurus*, *Blikanasaurus*, *Camelotia* and *Aardonyx* as well as the clade including *Antetonitrus*, *Lessemsaurus* and Sauropoda (Fig. 9A). This clade, which is sister to *Jingshanosaurus*, is diagnosed by the following unambiguous synapomorphies: longitudinal axis of the femur weakly bent with an offset of less than 10 degrees in lateral view (character 280, state 1), proximal tip of lesser trochanter distal to the femoral head (character 286, state 1), fourth trochanter along the length of the femur straddling the midpoint (character 293, state 1). Alternatively, the majority-rule consensus (Fig. 9B) resolves *Meroktenos* in a trichotomy with *Blikanasaurus* and the clade including *Melanorosaurus* and the other sauropodomorphs closer to Sauropoda, suggesting that *Meroktenos* and *Melanorosaurus* are not sister groups. The analysis was also run with just the femur scored. As a result, *Meroktenos* appears amongst Sauropoda, nested more deeply than the basal sauropods *Vulcanodon* and *Tazoudasaurus*, emphasizing the peculiar morphology of the femur in *Meroktenos*.

## Discussion

Many basal sauropodomorphs, including *Antetonitrus*, *Camelotia*, *Jingshanosaurus*, *Lessemsaurus*, *Melanorosaurus* and *Sarahsaurus* have been regarded as having a marked eccentricity of the femur (Apaldetti, Pol & Yates, 2013: character 281, state 1. See also Upchurch, Barrett & Galton, 2007; Yates et al., 2010; Pol, Garrido & Cerda, 2011). It appears that when we quantify this character (Table 3), *Meroktenos* has the highest eccentricity among non-sauropod sauropodomorphs, its value falling within the variation range of Sauropoda. This result is quite unexpected considering the small size of the femur of *Meroktenos* (Table 2). Indeed, it has been suggested that the high eccentricity of sauropod femora is size-related (Carrano, 2005). With the acquisition of larger body sizes in sauropods, eccentricity of the femur has been linked to the graviportalism of the group, and to an increasing resistance to mediolateral bending (Wilson & Carrano, 1999; Carrano, 2001). Based on the proportions of the ulna and radius of *Meroktenos* and their size relative to the femur, they do not seem adapted to support a quadrupedal gait, thus *Meroktenos* was most probably bipedal. *Antetonitrus*, depicted as a facultative biped (McPhee et al., 2014), is the only other non-sauropod sauropodomorph to present an eccentricity greater than 1.50 (Table 3). *Meroktenos* is the first Triassic ‘prosauropod’ with a ‘sauropod-like’ midshaft cross section of the femur and yet, a reduced size.

The relatively reduced size of the specimen could have indicated that we deal with a juvenile. In this case, the eccentricity and size of the femur might be temporary features. However, given our observations in several institutions, femora coming from juvenile specimens (less than 300 mm) rarely differ in eccentricity from adults femora of the same genus. Finally, an ontogenetic study including 5 sauropodomorphs, of which 2 basal sauropodomorphs, demonstrated that isomeric growth is ancestral for Dinosauria, and that the growth in the femora of sauropodomorphs was not significantly different from isometry (Kilbourne & Makovicky, 2010). Hence, assuming that the specimen from Thabana Morena is a juvenile (which is unlikely given its 480 mm length), its growth was isometric and the eccentricity of the femur was also present in adult forms of this taxon.

*Meroktenos* represents a completely new array of forms with anatomical features that were to become key adaptations to graviportalism and quadrupedalism, even before the body mass increase leading to huge sauropod dinosaurs.

## Conclusions

The anatomy of the basal sauropodomorph from Thabana Morena is enhanced by the rediscovery and description of additional postcranial remains belonging to the type femur, which had been incorrectly assigned to *Melanorosaurus*, as *M. thabanensis*, for the last 22 years. Through anatomical comparison combined with the first phylogenetic analysis including *Melanorosaurus* and *Meroktenos*, we showed that the Thabana Morena specimen can not be referred to *Melanorosaurus* and that the erection of a new genus was necessary. *Meroktenos thabanensis* comb. nov. is closely related to other basal sauropodomorphs often referred to as the sister group of Sauropoda. It is a Late Triassic form, increasing the number of Late Triassic basal sauropodomorphs worldwide to 26, seven of which come from Southern Africa. With four genera currently known in the Lower Jurassic of the same region, the paleobiodiversity of sauropodomorphs seems to decrease marginally after the Triassic-Jurassic boundary. Furthermore, we can now affirm that no Late Triassic basal sauropodomorph species survived beyond this boundary (*contra*Gauffre, 1993a; Galton & Upchurch, 2004). *Meroktenos* represents a new form in the growing number of Gondwanan basal sauropodomorphs closely related to Sauropoda. However, it is the only one known from Triassic deposits to exhibit a small ‘sauropod-like’ femur, and this new genus could be among the first basal sauropodomorphs to acquire key anatomical adaptations, some of which would be retained later in massive sauropod dinosaurs.

## Supplemental Information

10.7717/peerj.1639/supp-1 Supplemental Information 1 Phylogenetic matrix

## Institutional abbreviations

BP: Evolutionary Studies Institute, Johannesburg, South Africa (formerly Bernard Price Institute)
CM: Carnegie Museum of Natural History, Pittsburgh, Pennsylvania, USA
CPSGM: Collections Paléontologiques du Service Géologique du Maroc, Rabat, Morocco
MB: Museum für Naturkunde, Berlin, Germany
MNHN: Muséum National d’Histoire Naturelle, Paris, France
NM QR: National Museum, Bloemfontein, South Africa
OUMNH: Oxford University Museum of Natural History, Oxford, England
PVL: Universidad Nacional de Tucumán, San Miguel de Tucumán, Argentina
SAM-PK: Iziko South African Museum, Cape Town, South Africa
YPM: Yale Peabody Museum of Natural History, New Haven, CT, USA

## Additional Information and Declarations

### Data Availability

The following information was supplied regarding data availability: The data set used for the phylogenetic analysis is provided in the Supplemental Information.

### New Species Registration

The following information was supplied regarding the registration of a newly described species: Publication LSID: urn:lsid:zoobank.org:pub:6BD17539-F024-432A-9232-B220BBBE0EDF Meroktenos gen. nov. LSID: urn:lsid:zoobank.org:act:D2F95159-3806-4EF8-98DF-F027E5810C1D.
